# Default Mode Network Analysis of APOE Genotype in Cognitively Unimpaired Subjects Based on Persistent Homology

**DOI:** 10.3389/fnagi.2020.00188

**Published:** 2020-06-30

**Authors:** Liqun Kuang, Jiaying Jia, Deyu Zhao, Fengguang Xiong, Xie Han, Yalin Wang, for the Alzheimer’s Disease Neuroimaging Initiative

**Affiliations:** ^1^School of Data Science and Technology, North University of China, Taiyuan, China; ^2^School of Computing, Informatics, and Decision Systems Engineering, Arizona State University, Tempe, AZ, United States

**Keywords:** APOE, Alzheimer’s disease, persistent homology, resting state functional magnetic resonance imaging, graph theory, network measure

## Abstract

Current researches on default mode network (DMN) in normal elderly have mainly focused on finding some dysfunctional areas with decreased or increased connectivity. The global network dynamics of apolipoprotein E (APOE) e4 allele group is rarely studied. In our previous brain network study, we have demonstrated the advantage of persistent homology. It can distinguish robust and noisy topological features over multiscale nested networks, and the derived properties are more stable. In this study, for the first time we applied persistent homology to analyze APOE-related effects on whole-brain functional network. In our experiments, the risk allele group exhibited lower network radius and modularity in whole brain DMN based on graph theory, suggesting the abnormal organization structure. Moreover, two suggested measures from persistent homology detected significant differences between groups within the left hemisphere and in the whole brain in two datasets. They were more statistically sensitive to APOE genotypic differences than standard graph-based measures. In summary, we provide evidence that the e4 genotype leads to distinct DMN functional alterations in the early phases of Alzheimer’s disease using persistent homology approach. Our study offers a novel insight to explore potential biomarkers in healthy elderly populations carrying APOE e4 allele.

## Introduction

Alzheimer,s disease (AD) (Lane et al., 2018) is the most common form of dementia among the elderly and the sixth leading cause of death in the United States. There are more than 50 million patients worldwide in 2018, and it is expected to reach a staggering 152 million by 2050 (Patterson, 2018). It is crucial to develop the AD-related biomarkers early in the aging process before the onset of overt cognitive impairment and irreversible brain damage (Korthauer et al., 2018). One hypothesis (Reiman et al., 2009; Lambert et al., 2013; Yu et al., 2019) for the pathogenesis (Karch and Goate, 2015) of AD indicate the apolipoprotein E (APOE) e4 allele (Lane-Donovan and Herz, 2017) involves the accumulation of Amyloid-β (Caselli et al., 2010), leading to increasing neuronal atrophy and synapse loss. To date, APOE is a major genetic risk factor for developing AD (Thompson et al., 2013; Zhu et al., 2019). Functional neuroimaging genetics provides an effective strategy for characterizing the intermediate phenotype of AD and identifying genes that contribute to functional alterations in brain networks (Chiesa et al., 2017, Chiesa et al., 2019). In particular, recent research has demonstrated that default mode network (DMN) (Raichle, 2015) is associated with progressive brain dysfunction and is susceptible to APOE genotype (Song et al., 2015; Ma et al., 2016; Yuan et al., 2016; Palmqvist et al., 2017; Staffaroni et al., 2018; Chiesa et al., 2019).

Graph theory has increasingly been used as a theoretical framework for studying brain network characteristics. At the level of regional connection, functional connectivity between brain nodes as an important biomarker can identify early brain function alteration related to AD pathophysiology (Bokde et al., 2009). It is dedicated to investigating the distinct connectivity within the DMN that could represent the progressive biomarker. However, the results of resting state functional MRI (rs-fMRI) (Teipel et al., 2015) and APOE studies have reported mixed results (Cai et al., 2017; Luo et al., 2017; Caldwell et al., 2019; Zhu et al., 2019). Some reported decreased functional connectivity (Yan et al., 2015) in APOE e4 allele carriers (APOE4+) compared with non-carries (APOE4-), the others found some increased functional connectivity (Song et al., 2015; Zhu et al., 2018), while others didn’t found any differences (Chiesa et al., 2019). At the global whole-brain level, some neurobiologically meaningful graph-theoretic properties have become important indicators for measuring brain functional networks, through which we can understand the altered network architecture in those carrying risk genotype, including a loss of small-world network (Korthauer et al., 2018), a redistribution of hubs (Wink et al., 2018), and a disrupted modular organization (Li et al., 2019). However, there are currently few network measures based on graph theory have been studied in cognitively unimpaired elderly (Seo et al., 2013; Luo et al., 2017; Pietzuch et al., 2019) and some of their results were reported as inconsistent (Seo et al., 2013; Qiu et al., 2016; Luo et al., 2017). For instance, Wink et al. (2018) found decreased centrality of DMN in APOE4+ comparing to non-carriers, while Wang et al. (2017) didn’t find such genotype difference of centrality in normal elderly. Overall, graph-theoretic methods cannot consistently demonstrate functional DMN difference between APOE4+ and APOE4- in normal elderly, and the reason has been debated in the literature (Chiesa et al., 2017).

Recently, persistent homology (Edelsbrunner and Harer, 2010) from algebraic topology has been adopted for the analysis of brain network. It uses graph filtration to construct a multiscale brain network with all possible thresholds wherever the persistent topological features over the network dynamics are identified (Giusti et al., 2016). This method can distinguish robust and noisy topological features over a wide range of filtration values in measuring global brain network organization. The typical approach of persistent homology is Betti number plot (BNP) (Edelsbrunner and Harer, 2010; Lee et al., 2012), which has successfully applied to the brain network research on epilepsy (Choi et al., 2014), autism spectrum disorder and attention-deficit hyperactivity disorder (Lee et al., 2012, 2017), etc. In our previous works (Kuang et al., 2019a, b), we have developed some network properties based on persistent homology and have successfully applied them to measure the metabolic and functional networks of AD and MCI patients. Although the persistent homology works well in cognitively impaired elderly, it has never been applied to study the genetic influence on brain network yet, especially in unimpaired individuals.

In this paper, we study the effect of APOE genotype on functional DMN in cognitively unimpaired subjects. We hypothesized the topological properties of persistent homology may reveal the APOE-related alteration in DMN even before clinical symptoms appear better than graph-theoretic approaches. Using the cross-sectional rs-fMRI imaging data of 27 APOE4+ and 31 APOE4- normal elderly, we test this hypothesis by computing two persistent homology-based properties and measuring the differences between APOE4+ and APOE4- groups. We further run the statistical inference to validate their powers and compare them with some graph-theoretic methods.

## Materials and Methods

**FIGURE 1:**
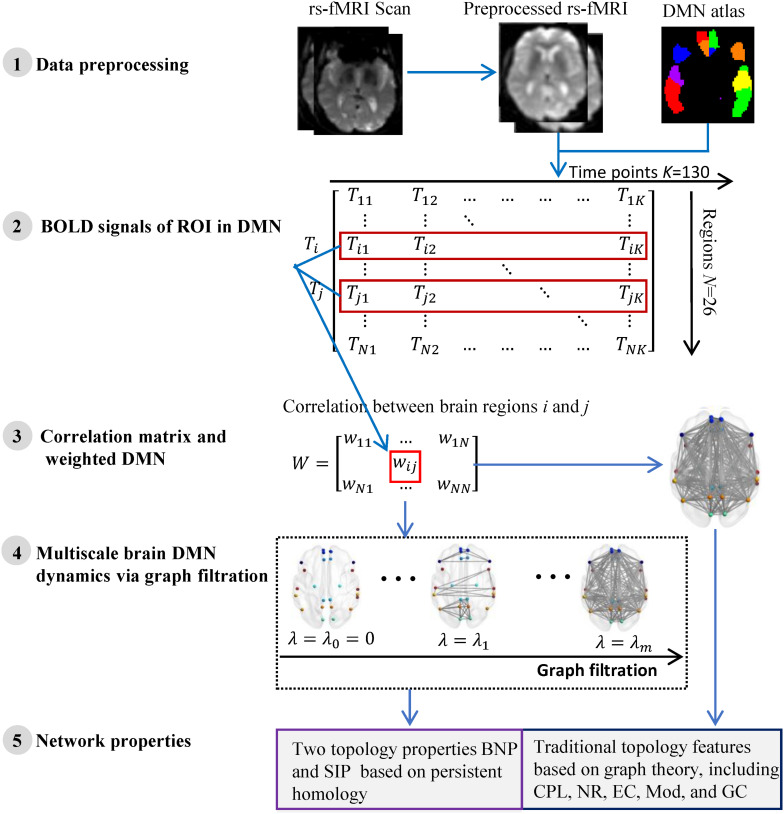
The flow of measuring DMN topological structure based on graph theory and persistent homology in cognitively unimpaired subjects using rs-fMRI data from ADNI.

We summarize the pipeline of our framework in Figure 1. The rs-fMRI (Teipel et al., 2015) data of each subject are preprocessed and the blood oxygen level dependent (BOLD) signals within each region-of-interest (ROI) are obtained. Then we construct one weighted DMN per subject and quantify its global topological structure using graph theory and persistent homology. The details are described in following subsections.

### Participants

Data used in the preparation of this article were obtained from the Alzheimer’s Disease Neuroimaging Initiative (ADNI) database^1^ (Jack et al., 2008; Jagust et al., 2010). The ADNI was launched in 2003 as a public-private partnership, led by Principal Investigator Michael W. Weiner, MD. The primary goal of ADNI has been to test whether serial magnetic resonance imaging (MRI), positron emission tomography (PET), other biological markers, and clinical and neuropsychological assessment can be combined to measure the progression of mild cognitive impairment (MCI) and early Alzheimer’s disease (AD).

There were only 38 Normal Controls (NC) between the ages of 60 and 90 from the ADNI-2 who had available rs-fMRI and APOE data. Due to the small sample size of NC, we also introduced Subjective Memory Complaints (SMC), producing a dataset of cognitively unimpaired subjects in this study. The only difference from NC is that SMC reported memory problems by themselves. Individuals with two copies of the apolipoprotein e2 allele (APOE e2/2) were excluded due to its possible protective effects (Suri et al., 2013). Finally, individuals carrying at least one APOE ε4 allele (genotype e3/e4 and e4/e4) were classified as APOE4+, while individuals with genotype e3/e3 were classified as APOE4-.

### Data Acquisition and Preprocessing

The experimental dataset were acquired at multiple ADNI sites using 3.0 T Philips MRI scanners. All rs-fMRI data were obtained using an echo-planar imaging (EPI) sequence and the parameters included repetition time (TR) = 3000 ms, echo time (TE) = 30 ms, flip angle = 80°, number of slices = 48, slice thickness = 3.3 mm, voxel size = 3 mm × 3 mm × 3 mm, voxel matrix = 64 × 64, and time points = 140.

All functional images were pre-processed using SPM8 toolbox^2^, DPARSF^3^ (Yan and Zang, 2010), and REST^3^ (Song et al., 2011) according to well-accepted pipelines, the same as our prior work (Kuang et al., 2019a). Briefly, the first ten time points were removed before temporal correction and spatial normalization. Then image smoothing, linear trend adjustment and band-pass filter were performed sequentially.

### Construction of DMN

First, the whole brain is divided into 90 functional ROI using standard automated anatomical labeling atlas (AAL90) (Tzourio-Mazoyer et al., 2002). Then 26 areas (Vriend et al., 2018) in AAL90 are identified as the ROI of DMN, as shown in Table 1. There are 13 ROI per hemisphere and each ROI is considered as a network node of DMN.

**TABLE 1:** The division of twenty six ROI nodes in DMN based on AAL90 atlas.

ROI node name	Left hemisphere		Right hemisphere	
	Index in AAL90	Abbreviation	Index in AAL90	Abbreviation
Inferior frontal gyrus pars triangularis	13	IFGtriang.L	14	IFGtriang.R
Medial frontal gyrus	23	SFGmed.L	24	SFGmed.R
Superior medial orbital frontal cortex	25	ORBsupmed.L	26	ORBsupmed.R
Anterior cingulate and paracingulate gyrus	31	ACG.L	32	ACG.R
Posterior cingulate gyrus	35	PCG.L	36	PCG.R
Parahippocampal gyrus	39	PHG.L	40	PHG.R
Cuneus	45	CUN.L	46	CUN.R
Supramarginal gyrus	63	SMG.L	64	SMG.R
Angular gyrus	65	ANG.L	66	ANG.R
Precuneus	67	PCUN.L	68	PCUN.R
Superior temporal gyrus	81	STG.L	82	STG.R
Temporal pole: superior temporal gyrus	83	TPOsup.L	84	TPOsup.R
Middle temporal gyrus	85	MTG.L	86	MTG.R

The average timing BOLD signal serial *T*_*i*_ = (*T*_*i*__1_, *T*_*i*__2_,…*T*_*ik*_) within the *i-th* ROI node is used as its measurement (Step 2 in Figure 1). We define the functional connectivity (i.e., edge weight) between any pair of ROI as 1-Pearson coefficient of their BOLD signal serials, i.e.

(1) $$\begin{array}{rcl} W_{ij} & = & 1-\frac{cov(T_{i},T_{j})}{\sigma_{Ti}\sigma_{Tj}}\\ & & =1-\frac{\sum_{p=1}^{k}(T_{ip}-{\bar{T}}_{i})(T_{jp}-{\bar{T}}_{j})}{\sqrt{\sum_{p=1}^{k}(T_{ip}-{\bar{T}}_{i}{)}^{2}}\sqrt{\sum_{p=1}^{k}(T_{jp}-{\bar{T}}_{j}{)}^{2}}} \end{array}$$

where *T*_*ip*_ represents the average BOLD signal within the *i-th* ROI at *p-th* time point and *K* = 130 is total number of time points of the rs-fMRI data. Thus, the functional connection matrix (*N* × *N*) per subject is obtained (Step 3 in Figure 1) and each subject’s DMN is constructed. Here *N* = 26 if the DMN of entire brain is studied, otherwise *N* = 13 if only one hemispheric DMN is studied.

### Measuring DMN Using Graph Theory

In the past decade, the neurobiologically meaningful network properties based on graph theory have become important indicators in measuring brain functional networks. We validate some widely used graph measures in this study (Right part of step 5 in Figure 1), including characteristic path length (CPL) (Li et al., 2019), global efficiency (GC) (Shu et al., 2015), network radius (NR) (Fujita et al., 2017), modularity (Mod) (Li et al., 2019), and eigenvector centrality (EC) (Luo et al., 2017). Briefly, the average shortest path length between all pairs of nodes in the network is CPL, while the average inverse shortest path length is called GC. Then NR is the minimum eccentricity of all nodes in the network and nodal eccentricity is the greatest distance between this node and any other nodes. Further, Mod measures the extent to which the network can be subdivided into clearly delineated and non-overlapping groups, and EC computes the sum of centralities of the node’s direct neighbors. All these network measures were calculated by Brain Connectivity Toolbox (BCT)^4^ in Matlab R2017a.

### Measuring DMN Using Persistent Homology

Persistent homology (Edelsbrunner and Harer, 2010) is a mathematical concept derived from algebraic topology and is used to characterize topological features in complex data. There is an important tool, graph filtration (Giusti et al., 2016), in persistent homology that constructs a family of nested networks along an axis at their threshold values by thresholding original weighted network at every possible entry (Step 4 in Figure 1). Thus, it can distinguish robust and noisy topological characteristics in a wide range and enables reasonable inferences regarding the underlying organization. The classic network property based on persistent homology is BNP which detects the dynamic of the zeroth Betti number (i.e., the number of connected components) over all filtration values (Lee et al., 2012). It has been successfully applied to the some studies (Lee et al., 2012, 2017; Choi et al., 2014) of brain network in neurodegenerative diseases.

In our previous work (Kuang et al., 2019a), we proposed an integrated persistent feature (IPF) based on BNP, which introduced a connected component aggregation cost into the zeroth Betti number and thus achieves a holistic description of network dynamics. The IPF at filtration λ*_*i*_* is defined as (Kuang et al., 2019a).

(2) $$IPF\lambda_{i}=\left\{ \begin{array}{cc} \begin{array}{c} \frac{m-i}{m\left(m-1\right)}\sum_{k=i+1}^{m-1}\lambda_{k} \end{array} & 0\leq i\leq m-2\\ 0 & i=m-1 \end{array}\right.$$

Here, *m* is total number of network nodes and λ_0_ = 0 < λ_0_ < λ_1_ < λ_2_ < … < λ*_*m*_*_–1_ is the filtration value which is actually the set of weights of minimum spanning tree of the original weighted network. Previous work has proven that the IPF is a monotonically decreasing convergence function over all possible filtration. In summary, when λ increases from zero, the IPF value of the network will decrease to zero accordingly until all nodes are connected into a single connected component. Therefore, the slope of the IPF plot (SIP) can be used as an important network property to quantify the brain network dynamics. Both network measures BNP and SIP can be considered as information diffusion rate or convergence rate of the network. We provided their implementations at http://gsl.lab.asu.edu/software/IPF and applied them in this study (Left part of step 5 in Figure 1).

## Results

### Demographic Information

In this experiment, 58 subjects without cognitive impairment were selected from ADNI-2, and were divided into two groups, APOE e4 carriers and non-carriers, according to their APOE genotype. Among them, 20 subjects were identified as SMC and remaining 38 subjects were NC. The only difference from NC is that SMC reported memory problems by themselves. We considered both as cognitively impairment subjects in this study. As shown in Table 2, there were no significant differences in age, education, Mini Mental State Examination (MMSE) score, and Clinical Dementia Rating (CDR) global scores between groups. All subjects had MMSE of 24–30, CDR = 0, and were cognitively unimpaired. Furthermore, all studied images did not have excessive head motion (six-parameter rigid body) defined by a displacement of less than 1 mm or an angular rotation of less than in any direction 1°.

**TABLE 2:** Demographic characteristics of the high-risk (APOE4+) and low-risk (APOE4-) groups.

	APOE4+ (*n* = 27)	APOE4- (*n* = 31)	*p*-value
NC/SMC	16/11	22/9	–
Age	73.26 ± 6.83	74.58 ± 5.18	0.407
Education	16.81 ± 2.17	17.22 ± 2.93	0.569
Male/Female	12/15	15/16	0.769
MMSE Score	28.17 ± 1.53	28.53 ± 1.55	0.734

*Data is presented as means ± standard deviations. APOE4+, APOE e3/e4 and e4/e4 alleles; APOE4-: APOE e3/e3 allele; NC, Normal Control; SMC, Subjective Memory Complaint; MMSE, Mini-Mental State Examination; CDR, Clinical Dementia Rating.*

### Multiscale Brain DMN Dynamics

**FIGURE 2:**
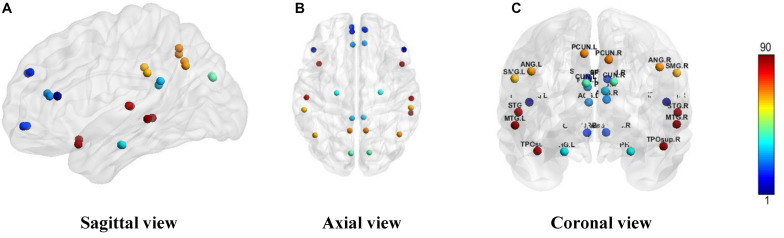
The ROI in DMN from **(A)** sagittal view, **(B)** axial view, and **(C)** coronal view. The color bar shows the ROI node index predefined in AAL90 atlas.

**FIGURE 3:**
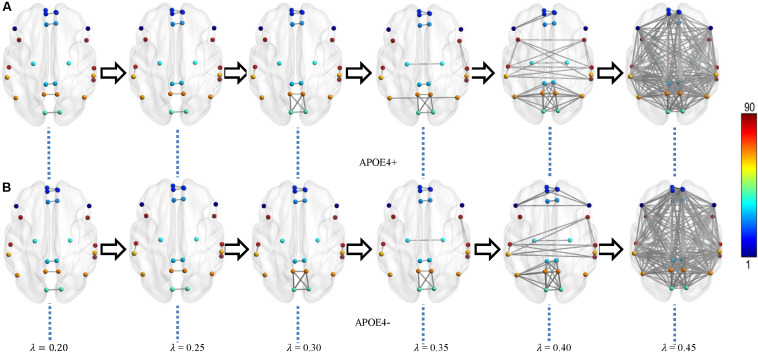
Multiscale DMN dynamics for two mean networks of **(A)** APOE4+ and **(B)** APOE4- groups at six different filtration values 0.20, 0.25, 0.30, …, 0.45. The color bar shows the ROI node index predefined in AAL90 atlas.

**FIGURE 4:**
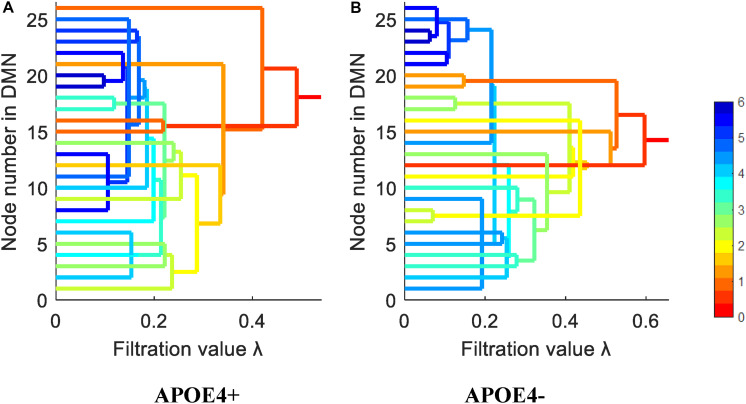
The single linkage dendrograms of **(A)** APOE4 carriers and **(B)** non-carriers groups show the change of the zeroth Betti number. The color represents the target distance (total edge weight) from current connected component to the full connected component (the rightmost line).

We constructed one original weighted DMN per subject. The 26 network nodes in whole brain DMN were determined according to Table 2 and are visualized in Figure 2 using Brain Net Viewer software (Xia et al., 2013). Then, the edge weights between them were calculated using Eq. (1). We further constructed multiscale networks based on the original DMN using graph filtration tool (Step 4 in Figure 1). As we only observe the zeroth homology in this study, the filtration value λ is actually the set of weights of minimum spanning tree of DMN. Figure 3 shows the multiscale network dynamics for two mean DMN of two groups over some filtration values. Figure 4 shows the change of zeroth Betti number using single linkage dendrogram (Lee et al., 2012). All the nodes on the left are connected to form the larger component on the right, until finally a fully connected network is constructed. The zeroth Betti number starts at 26, and gradually decreases to 1 while more and more nodes are connected.

From Figures 3, 4, we intuitively saw that the connected components in APOE+ aggregated slightly faster than APOE-, especially after λ is larger than 0.35. However, it needs to be further quantitatively measured by network properties based on persistent homology.

### Brain DMN Properties

**FIGURE 5:**
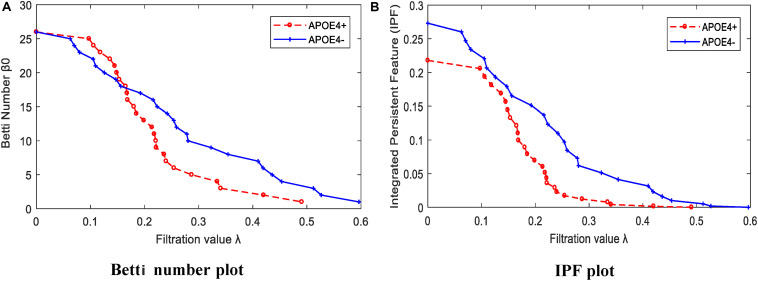
The persistent feature dynamics over filtrations for APOE4+ and APOE4- by **(A)** Betti number plot and **(B)** IPF plot.

**FIGURE 6:**
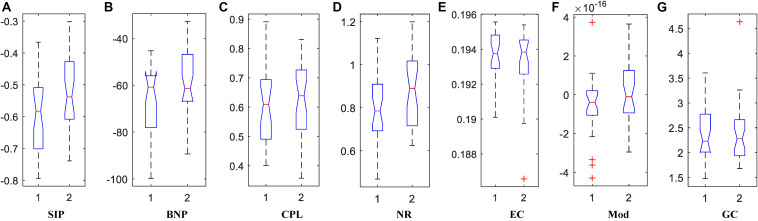
The box plots of property values for APOE4 carriers (1) and non-carriers (2) groups using two persistent homology properties, **(A)** SIP and **(B)** BNP, and five graph-theoretic properties, **(C)** CPL, **(D)** NR, **(E)** EC, **(F)** Mod, and **(G)** GC.

We calculated the corresponding Betti number β_0_ and IPF of the multiscale DMN at all different filtration values for two group means, and plotted them, as shown in Figure 5. We found that the APOE+ curve in both Betti number plot and IPF plot were steeper than the APOE- curve, suggesting the faster aggregation of APOE+, which is consistent with the above observation of multiscale brain dynamics (see Figures 3, 4). All subjects’ values of BNP and SIP properties based on persistent homology were summarized using box plot as shown in Figures 6A,B, separately, where 1 represents APOE4+ and 2 is APOE4-. The distributions of both BNP and SIP property values between groups are obviously different, indicating both persistent features may be able to discriminate APOE4+ from APOE4-.

Traditionally, brain network properties have been measured using graph theory methods. In order to compare with our suggested methods based on persistent homology, we also calculated some classical graph theory properties, including CPL, NR, EC, Mod, and GC. The distributions of all attribute values are shown in Figures 6C–G where 1 and 2 represents APOE4+ and APOE4-, respectively. We observed that the between-group differences of SIP, BNP, NR, and Mod are more apparent than those of CPL, EC, and GC.

### Statistical Group Difference

In the statistical analysis of differences between groups of APOE4+ and APOE4-, we performed the permutation test of 10,000 permutations on all network properties using Matlab R2017a and calculated their resulting *p*-values as shown in Table 3. First, the differences between groups in whole brain DMN with 26 ROI nodes were measured. Two persistent features SIP and BNP obtained significant differences at significance level of 0.05, which were *p* = 0.021 and *p* = 0.009, respectively. In the statistic inferences for five compared graph theory-based properties, only NR and Mod obtained significant differences with *p* = 0.024 and *p* = 0.037, respectively, while there were no significant differences in other three properties, CPL, EC, and GC. Then, we analyzed the group differences within the single hemisphere and only two measures SIP and BNP achieved significant differences (*p* = 0.027, both) within the left hemisphere. We did not find any differences within the right hemisphere.

**TABLE 3:** Statistical *p*-values of different network properties between APOE4+ and APOE4- groups.

Hemisphere	Persistent homology-based properties		Graph theory-based properties				
	SIP	BNP	CPL	NR	EC	Mod	GC
Both	0.021	0.009	0.237	0.024	0.273	0.037	0.458
Left	0.027	0.027	0.316	0.154	0.422	0.361	0.418
Right	0.144	0.097	0.134	0.052	0.281	0.090	0.393

*The permutation test of 10,000 permutations was performed for statistical inference. APOE4+, APOE e3/e4 and e4/e4 alleles; APOE4-: APOE e3/e3 allele; SIP, Slope of Integrated persistent feature Plot; BNP, Betti Number Plot; CPL, Characteristic Path Length; NR, Network Radius; EC, Eigenvector Centrality; Mod, modularity; GC, Global Efficiency.*

In short, our experimental results show that both persistent properties achieved more significant group differences between APOE4+ and APOE4- than traditional graph-theoretic measures, and BNP obtained the most significant difference (*p* = 0.009) in the study of whole brain DMN.

## Discussion

### Present Findings

There are three main findings in this study.

First, we found that the e4 allele carriers exhibited lower NR and Mod (*p* = 0.024, 0.037, respectively) in the study of whole brain DMN using traditional graph-theoretic methods, suggesting the abnormal organization structure in the risk allele group. To our knowledge, there have been few studies (Luo et al., 2017; Pietzuch et al., 2019) on graph theory that have reported APOE genotypic differences in functional network properties of whole brain DMN in normal elderly, although a lot of studies have found differences in functional network properties between AD/MCI and NC. Some studies (Staffaroni et al., 2018; Chiesa et al., 2019) even found no difference between elderly APOE4+ and APOE4- groups in functional DMN. The reason why findings of DMN on unimpaired individuals do not consistently demonstrate differences between APOE4+ and APOE4- is still debated in the literature (Chiesa et al., 2017). In our study, we found two measures could detect their differences significantly, which would further enhance the APOE research based on graph theory.

Second, we introduced two measures from our previous studies based on persistent homology and found they were more statistically powerful than graph-theoretic measures in discriminating APOE4+ from APOE4- in our experiment, and the BNP obtained the most significant difference (*p* = 0.009) between groups. The persistent homology approach can distinguish robust and noisy topological features over multiscale nested networks, and the obtained properties are more persistent and stable. So far, many studies of brain network based on persistent homology have demonstrated the superiority of the performance. To our knowledge, it is the first time we introduced persistent homology to study the APOE genotype effect on DMN.

Finally, the functional disruption within the left hemisphere may be more pronounced than the right one. All persistent homology-based features SIP and BNP detected the significant differences of DMN in whole brain and left hemisphere. However, no significant differences were found within the right hemispheric DMN using any network measures. This finding is consistent with existing APOE studies documenting the effect of the e4 allele on left hippocampus rather than right hippocampus of non-demented individuals (Shi et al., 2014; Li et al., 2016; Dong et al., 2019).

### Verification on Normal Control Subjects

As there were only 16 NC individuals who had e4 allele and available rs-fMRI data in ADNI-2, we expanded our experimental sample size by including SMC subjects. However, some studies (Caldwell et al., 2019) on APOE have only investigated NC individuals from ADNI. Thus, we excluded all SMC subjects (see Table 2) and further repeated our experiment on NC subjects. As shown in Table 4, there were no significant differences in age, education, sex, and MMSE between APOE+ and APOE- in NC dataset. We calculated the differences of DMN between groups using different measures, as shown in Table 5. Again, two measures based on persistent homology obtained stronger statistical power than graph theory methods, and the differences within the left hemispheric DMN is more significant than the right one. Moreover, compared to experiments performed on cognitively unimpaired dataset (Table 3), the SIP obtained very significant difference (*p* = 0.006) on NC dataset.

**TABLE 4:** Demographic characteristics of NC dataset.

	APOE4+ (*n* = 16)	APOE4- (*n* = 22)	*p*-value
Age	73.88 ± 7.35	75.75 ± 5.34	0.368
Education	16.53 ± 1.97	16.84 ± 2.84	0.462
Male/Female	7/9	10/12	0.920
MMSE Score	28.59 ± 1.65	28.77 ± 1.66	0.833

*Data is presented as means ± standard deviations. APOE4+, APOE e3/e4 and e4/e4 alleles; APOE4-, APOE e3/e3 allele; NC, Normal Control; MMSE, Mini-Mental State Examination; CDR, Clinical Dementia Rating.*

**TABLE 5:** Statistical *p*-values of different network properties on NC between APOE4+ and APOE4- groups.

Hemisphere	Persistent homology-based properties		Graph theory-based properties				
	SIP	BNP	CPL	NR	EC	Mod	GC
Both	0.006	0.004	0.365	0.062	0.183	0.037	0.201
Left	0.047	0.049	0.364	0.177	0.276	0.089	0.368
Right	0.078	0.098	0.190	0.095	0.162	0.195	0.352

*APOE4+, APOE e3/e4 and e4/e4 alleles; APOE4-: APOE e3/e3 allele; SIP, Slope of Integrated persistent feature Plot; BNP, Betty Number Plot; CPL, Characteristic Path Length; NR, Network Radius; EC, Eigenvector Centrality; Mod, modularity; GC, Global Efficiency.*

### Limitation and Future Works

Despite the promising results were obtained by applying two suggested network properties SIP and BNP based on persistent homology to discriminate APOE e4 allele carriers from non-carriers in cognitively unimpaired subjects, there are three important caveats.

First, both persistent homology-based properties BNP and SIP adopted in this study only investigated the dynamics of the zeroth persistent homology. Higher-dimensional persistent homology characterizes higher-dimensional topological features, and more complexed topological structures such as circular holes can be detected. Therefore, the performance of network measurement may be further boosted if higher-dimensional homology is applied, especially in the sparse network that tends to have more holes.

Then, although the DMN have been heavily studied and are reported as a promising kind of network to study, current researches have mainly focused on finding some dysfunctional areas with decreased or increased connectivity (Song et al., 2015; Yan et al., 2015; Zhu et al., 2018; Chiesa et al., 2019). There are relatively few studies on global network dynamics of e4 allele group in normal elderly. In this study, we measured the global brain network in two datasets (cognitively unimpaired dataset and its subset NC) and found some statistically powerful measures. In future, we will validate these measures in other independent datasets.

Finally, current findings are achieved based on cross-sectional study. With longitudinal analysis, we may further study the longitudinal trajectories (Chiesa et al., 2019) of functional brain dynamics and the impact of e4 allele on individuals at risk for Alzheimer’s disease by quantifying the difference of their persistent features. In addition, the more aggregated structure in APOE e4 allele carriers may be due to worse development in childhood. Examining longitudinal MRI since childhood, the possibility of this phenomenon could be further assessed.

## Conclusion

This work measured the DMN structure of rs-fMRI on cognitively unimpaired e4 allele carriers based on our prior work of persistent homology, which encodes a great deal of dynamic information over all possible scales. The significant differences between APOE4+ and APOE4- are identified within the left hemispheric DMN and in the whole brain DMN in two datasets, providing evidence that the APOE e4 genotype leads to distinct alterations of functional DMN several years before the occurrence of dementia symptoms. Moreover, our suggested approaches of persistent homology are more sensitive to APOE genotypic differences than standard graph-based network measures. To the best of our knowledge, this is the first study applying persistent homology to analyze APOE-related effect on whole-brain functional network. This study offers a novel insight to explore potential biomarkers in healthy elderly populations carrying APOE e4 allele.

## Data Availability Statement

Publicly available datasets were analyzed in this study. This data can be found from the ADNI (http://adni.loni.usc.edu).

## Ethics Statement

In this study, all subjects were selected from the Alzheimer’s Disease Neuroimaging Initiative (ADNI) database (http://adni.loni.usc.edu/). All ADNI subjects gave written informed consent at enrollment for data collection, storage, and use for research.

## Author Contributions

LK and YW designed the study and revised the manuscript. FX acquired the data. JJ, DZ, and LK analyzed and interpreted the results of the data. LK and XH drafted the manuscript. All authors contributed to the article and approved the submitted version.

## Conflict of Interest

The authors declare that the research was conducted in the absence of any commercial or financial relationships that could be construed as a potential conflict of interest.

## Footnotes

1 adni.loni.usc.edu

2 http://www.fil.ion.ucl.ac.uk/spm/

3 http://www.restfmri.net

4 https://sites.google.com/site/bctnet
